# Supplementary material for: Monocyte-derived transcriptome signature indicates antibody-dependent cellular phagocytosis as a potential mechanism of vaccine-induced protection against HIV-1
Source: eLife. 2021 Sep 17;10:e69577. doi: 10.7554/eLife.69577 (PMC8514236; doi:10.7554/eLife.69577)
Supplement: Source code 1. [file elife-69577-supp2.zip › source_code.html]

Monocyte-derived transcriptome signature indicates antibody-dependent cellular phagocytosis as a potential mechanism of vaccine-induced protection against HIV-1


Code 

- Show All Code
- Hide All Code

# Monocyte-derived transcriptome signature indicates antibody-dependent cellular phagocytosis as a potential mechanism of vaccine-induced protection against HIV-1

- 1. Source data and scripts
- 2. Environment
- 3. Composite gene expression scores (GES) are higher in the uninfected compared to infected groups
  - 3.1. Fig. 1A - D
- 4. GES is a stronger correlate of reduced risk of infection in RV144
  - 4.1. Fig. 2A
  - 4.2. Fig. 2B
  - 4.3. Fig. 2C
  - 4.4. Fig. 2D
- 5. Strong relationship between functional ADCP responses in a human vaccine trial and the protective RV144 signature
  - 5.1. Fig. 3A
  - 5.2. Fig. 3B
  - 5.3. Fig. 3C
  - 5.4. Fig. 3D
- 6. Overlapping enriched genes associating with ADCP response
  - 6.1. Fig. 4A
  - 6.2. Fig. 4B
  - 6.3. Fig. 4C
  - 6.4. Fig. 4D
- 7. Pathway analyses of the enriched genes in the different vaccine studies
  - 7.1. Fig. 5A & B
  - 7.2. Fig. 5C
- 8. Cellular origin of the RV144 signature
  - 8.1. Fig. 6A
  - 8.2. Fig. 6B
  - 8.3. Fig. 6C
  - 8.4. Fig. 6D
  - 8.5. Fig. 6E

### 1. Source data and scripts

Original and processed data and scripts are available at: https://figshare.com/s/3068444ff317d820680f

### 2. Environment

```
library(osDesign)
library(gplots)
library(rockchalk)
library(varhandle)
library(lattice)
library(ggplot2)
library(knitr)
library(forestplot)
library(survival)
library(fmsb)
library(InformationValue)
library(caret)
library(verification)
library(ROCR)
library(pROC)
library(plyr)
library(extrafont)
library(tidyverse)
library(cowplot)
library(magrittr)
library(huxtable)
library(Seurat)
library(ggvenn)
library(dplyr)
library(readxl)
library(pheatmap)
library(ggplotify)

opts_chunk$set(tidy.opts=list(width.cutoff=55),tidy=TRUE)

load("Data/RV144 GES Analysis.RData")
flrstatus <- ifelse(mydata_rv144_vac_enriched63_170$infect=="Yes",1,0)
sex <-ifelse(mydata_rv144_vac_enriched63_170$dem_sex=="Female",1,0)
risk.cat <-ifelse(mydata_rv144_vac_enriched63_170$BRA_risk=="Low",0,ifelse(mydata_rv144_vac_enriched63_170$BRA_risk=="Medium",1,2)) 
risk.medium <- ifelse(mydata_rv144_vac_enriched63_170$BRA_risk=="Medium",1,0)
risk.high <- ifelse(mydata_rv144_vac_enriched63_170$BRA_risk=="High",1,0)
EIGEN1 <-  mydata_rv144_vac_enriched63_170$EIGEN1
# making GES using scaled gene expression data (the enriched 63 genes)
trans63= rowMeans(mydata_rv144_vac_enriched63_170[,64:126])

####################### two phase design ##############################

nn0 <- c(length(dat1$pin[dat1$infect=="No" & dat1$cc_cohort==1 & dat1$trt=="VACCINE" & dat1$dem_sex=="Female" & dat1$perprot=="Yes" & dat1$vaccno==4]),
         length(dat1$pin[dat1$infect=="No" & dat1$cc_cohort==1 & dat1$trt=="VACCINE" & dat1$dem_sex=="Male" & dat1$perprot=="Yes" & dat1$vaccno==4]),
         length(dat1$pin[dat1$infect=="No" & dat1$cc_cohort==1 & dat1$trt=="VACCINE" & dat1$dem_sex=="Female" & dat1$perprot=="No" & dat1$vaccno==4]),
         length(dat1$pin[dat1$infect=="No" & dat1$cc_cohort==1 & dat1$trt=="VACCINE" & dat1$dem_sex=="Male" & dat1$perprot=="No" & dat1$vaccno==4]),
         length(dat1$pin[dat1$infect=="No" & dat1$cc_cohort==1 & dat1$trt=="VACCINE" & dat1$dem_sex=="Female" & dat1$perprot=="No" & dat1$vaccno<4]))

nn1 <- c(length(dat1$pin[dat1$infect=="Yes" & dat1$cc_cohort==1 & dat1$trt=="VACCINE" & dat1$dem_sex=="Female" & dat1$perprot=="Yes" & dat1$vaccno==4]),
         length(dat1$pin[dat1$infect=="Yes" & dat1$cc_cohort==1 & dat1$trt=="VACCINE" & dat1$dem_sex=="Male" & dat1$perprot=="Yes" & dat1$vaccno==4]),
         length(dat1$pin[dat1$infect=="Yes" & dat1$cc_cohort==1 & dat1$trt=="VACCINE" & dat1$dem_sex=="Female" & dat1$perprot=="No" & dat1$vaccno==4]),
         length(dat1$pin[dat1$infect=="Yes" & dat1$cc_cohort==1 & dat1$trt=="VACCINE" & dat1$dem_sex=="Male" & dat1$perprot=="No" & dat1$vaccno==4]),
         length(dat1$pin[dat1$infect=="Yes" & dat1$cc_cohort==1 & dat1$trt=="VACCINE" & dat1$dem_sex=="Female" & dat1$perprot=="No" & dat1$vaccno<4]))


stratuminds <- ifelse(mydata_rv144_vac_enriched63_170$dem_sex=="Female" & mydata_rv144_vac_enriched63_170$perprot=="Yes" & mydata_rv144_vac_enriched63_170$vaccno==4,1,
                      ifelse(mydata_rv144_vac_enriched63_170$dem_sex=="Male" & mydata_rv144_vac_enriched63_170$perprot=="Yes" & mydata_rv144_vac_enriched63_170$vaccno==4,2,
                             ifelse(mydata_rv144_vac_enriched63_170$dem_sex=="Female" & mydata_rv144_vac_enriched63_170$perprot=="No" & mydata_rv144_vac_enriched63_170$vaccno==4,3,
                                    ifelse(mydata_rv144_vac_enriched63_170$dem_sex=="Male" & mydata_rv144_vac_enriched63_170$perprot=="No" & mydata_rv144_vac_enriched63_170$vaccno==4,4,5))))
```

### 3. Composite gene expression scores (GES) are higher in the uninfected compared to infected groups

#### 3.1. Fig. 1A - D

Comparison of the GES between infected and uninfected groups of vaccinated NHP or humans. Mann-Whitney test or t test were used where assumptions were met (Figures 1A-D).

Source data for the dot plots: Fig1A.txt, Fig1B.txt, Fig1C.txt, Fig1D.txt

### 4. GES is a stronger correlate of reduced risk of infection in RV144

#### 4.1. Fig. 2A

A GES of the 63 enriched genes in the RV144 study was examined as a continuous variable. It associated with lower odds of HIV acquisition compared to the other two primary correlates of risk. Variables were measured at week 26, two weeks post last vaccination. For each variable, the odds ratio is reported per 1-SD increase.

```
scaled_trans63 = scale(trans63)
fit.tps <- tps(flrstatus ~ scaled_trans63 + EIGEN1 + sex + 
    risk.medium + risk.high, nn0 = nn0, nn1 = nn1, group = stratuminds, 
    method = "ML", cohort = TRUE)

x <- as.matrix(cbind(round(exp(fit.tps$coef[2]), 2), round(exp(fit.tps$coef[2] - 
    sqrt(fit.tps$covm[2, 2]) * 1.96), 2), round(exp(fit.tps$coef[2] + 
    sqrt(fit.tps$covm[2, 2]) * 1.96), 2), round(min(2 * 
    (1 - pnorm(abs(fit.tps$coef[2]/sqrt(fit.tps$covm[2, 
        2])))), 1), 4)))
colnames(x) = c("OR", "95%CI LB", "95%CI UB", "p value")
rownames(x) = "GES"
x
```

The OR of GES is plotted in a Forest plot with previous findings (HIV-1 specific IgG and IgA)

```
myforest=matrix(NA, 3, 4)
rownames(myforest)=c("GES","gp70-V1V2 (IgG)", "IgA (Env)")

myforest=as.data.frame(myforest)
colnames(myforest)=c("OR","95%CI LB","95%CI UB","p value")
myforest[1,]=c(0.37,0.22,0.63,"0.0002")
myforest[2,]=c(0.7,0.49,1.02,0.06)
myforest[3,]=c(1.39,1.00,1.93,0.05)


tabletext <- cbind(c("Immune Response",rownames(myforest)),
                   
                   c("p value",myforest$`p value`))


forestplot(labeltext=tabletext, graph.pos=2, 
           mean=c(NA,myforest$OR), 
           lower=c(NA,myforest$`95%CI LB`), upper=c(NA,myforest$`95%CI UB`),
        
           txt_gp=fpTxtGp(label=gpar(cex=0.7),
                          ticks=gpar(cex=0.5),
                          xlab=gpar(cex = 0.7),
                          title=gpar(cex = 1.0)),
           col=fpColors(box="black", lines="black", zero = "gray60"),
           zero=1,  lineheight = "auto", boxsize=0.11, colgap=unit(2.5,"mm"),
           lwd.ci=2, ci.vertices=TRUE, ci.vertices.height = 0.05,xticks = c(0,0.25,0.5,0.75,1,1.25, 1.5, 1.75, 2.00), xlab="\n Odds Ratio")
```

#### 4.2. Fig. 2B

Cumulative HIV-1 incidence curves were plotted for the three subgroups of vaccine recipients defined by tertiles into the lower, middle, and upper third of the GES (Low, Medium, High subgroups), as well as for the entire placebo group HIV negative at week 24 (n=6267 subjects) for reference. These curves were estimated using the Kaplan-Meier method with inverse probability weighting to account for the sampling design.

```
############## Cumulative Incidence Curve ##################### GES63
############## low med high #########

alpha1.0 <- length(unique(mydata_rv144_vac_enriched63_170$pin[mydata_rv144_vac_enriched63_170$trt == 
    "VACCINE" & mydata_rv144_vac_enriched63_170$infect == 
    "No" & mydata_rv144_vac_enriched63_170$dem_sex == "Female" & 
    mydata_rv144_vac_enriched63_170$perprot == "Yes" & mydata_rv144_vac_enriched63_170$vaccno == 
    4]))/length(dat1$pin[dat1$trt == "VACCINE" & dat1$infect == 
    "No" & dat1$cc_cohort == 1 & dat1$dem_sex == "Female" & 
    dat1$perprot == "Yes" & dat1$vaccno == 4])

alpha2.0 <- length(unique(mydata_rv144_vac_enriched63_170$pin[mydata_rv144_vac_enriched63_170$trt == 
    "VACCINE" & mydata_rv144_vac_enriched63_170$infect == 
    "No" & mydata_rv144_vac_enriched63_170$dem_sex == "Male" & 
    mydata_rv144_vac_enriched63_170$perprot == "Yes" & mydata_rv144_vac_enriched63_170$vaccno == 
    4]))/length(dat1$pin[dat1$trt == "VACCINE" & dat1$infect == 
    "No" & dat1$cc_cohort == 1 & dat1$dem_sex == "Male" & 
    dat1$perprot == "Yes" & dat1$vaccno == 4])

alpha3.0 <- length(unique(mydata_rv144_vac_enriched63_170$pin[mydata_rv144_vac_enriched63_170$trt == 
    "VACCINE" & mydata_rv144_vac_enriched63_170$infect == 
    "No" & mydata_rv144_vac_enriched63_170$dem_sex == "Female" & 
    mydata_rv144_vac_enriched63_170$perprot == "No" & mydata_rv144_vac_enriched63_170$vaccno == 
    4]))/length(dat1$pin[dat1$trt == "VACCINE" & dat1$infect == 
    "No" & dat1$cc_cohort == 1 & dat1$dem_sex == "Female" & 
    dat1$perprot == "No" & dat1$vaccno == 4])

alpha4.0 <- length(unique(mydata_rv144_vac_enriched63_170$pin[mydata_rv144_vac_enriched63_170$trt == 
    "VACCINE" & mydata_rv144_vac_enriched63_170$infect == 
    "No" & mydata_rv144_vac_enriched63_170$dem_sex == "Male" & 
    mydata_rv144_vac_enriched63_170$perprot == "No" & mydata_rv144_vac_enriched63_170$vaccno == 
    4]))/length(dat1$pin[dat1$trt == "VACCINE" & dat1$infect == 
    "No" & dat1$cc_cohort == 1 & dat1$dem_sex == "Male" & 
    dat1$perprot == "No" & dat1$vaccno == 4])

alpha5.0 <- length(unique(mydata_rv144_vac_enriched63_170$pin[mydata_rv144_vac_enriched63_170$trt == 
    "VACCINE" & mydata_rv144_vac_enriched63_170$infect == 
    "No" & mydata_rv144_vac_enriched63_170$dem_sex == "Female" & 
    mydata_rv144_vac_enriched63_170$perprot == "No" & mydata_rv144_vac_enriched63_170$vaccno < 
    4]))/length(dat1$pin[dat1$trt == "VACCINE" & dat1$infect == 
    "No" & dat1$cc_cohort == 1 & dat1$dem_sex == "Female" & 
    dat1$perprot == "No" & dat1$vaccno < 4])


cat63_3.vp <- as.factor(dat.vcc.allplc_3$cat63_3.vp)
flrtime.vp <- dat.vcc.allplc_3$time_to_infect - dat.vcc.allplc_3$time_to_wk26
flrstatus.vp <- ifelse(dat.vcc.allplc_3$infect == "Yes", 
    1, 0)
cat63.vp <- dat.vcc.allplc_3$cat63.vp
IGG.vp <- dat.vcc.allplc_3$IGG.vp
IGG_DPB13.vp <- dat.vcc.allplc_3$IGG_DPB13.vp
DPB13.vp <- dat.vcc.allplc_3$DPB13.vp


wt <- ifelse(flrstatus.vp == 1 | dat.vcc.allplc_3$trt == 
    "PLACEBO", 1, ifelse(dat.vcc.allplc_3$trt == "VACCINE" & 
    flrstatus.vp == 0 & dat.vcc.allplc_3$dem_sex == "Female" & 
    dat.vcc.allplc_3$perprot == "Yes" & dat.vcc.allplc_3$vaccno == 
    4, 1/alpha1.0, ifelse(dat.vcc.allplc_3$trt == "VACCINE" & 
    flrstatus.vp == 0 & dat.vcc.allplc_3$dem_sex == "Male" & 
    dat.vcc.allplc_3$perprot == "Yes" & dat.vcc.allplc_3$vaccno == 
    4, 1/alpha2.0, ifelse(dat.vcc.allplc_3$trt == "VACCINE" & 
    flrstatus.vp == 0 & dat.vcc.allplc_3$dem_sex == "Female" & 
    dat.vcc.allplc_3$perprot == "No" & dat.vcc.allplc_3$vaccno == 
    4, 1/alpha3.0, ifelse(dat.vcc.allplc_3$trt == "VACCINE" & 
    flrstatus.vp == 0 & dat.vcc.allplc_3$dem_sex == "Male" & 
    dat.vcc.allplc_3$perprot == "No" & dat.vcc.allplc_3$vaccno == 
    4, 1/alpha4.0, 1/alpha5.0)))))


################################################### KM plots by GES (low vs. med vs. high)
output.legend <- NULL
scl <- 365.25/12
new <- cat63_3.vp
levels(new) = c("0", "3", "1", "2")
new = factor(new, levels(new)[c(1, 3, 4, 2)])
V1 = new


par(mfrow = c(1, 1), mar = c(5, 4, 2, 3), las = 1, cex.axis = 1.1, 
    cex.lab = 1.1, cex.main = 1.1, oma = c(4, 2, 0, 0))
KM <- survfit(Surv(flrtime.vp/scl, flrstatus.vp) ~ V1, type = "kaplan-meier", 
    weights = wt)
sKM <- summary(KM, times = c(0, 12, 24, 36, 48), extend = T)
n.events.total <- tapply(sKM$n.event, rep(1:5, each = 4), 
    sum)
n.event <- matrix(round(sKM$n.event), nrow = 4, byrow = T)
n.atrisk <- matrix(round(sKM$n.risk), nrow = 4, byrow = T)
ptime <- unique(sKM$time)
output.legend <- data.frame(Variable = rep("GES63", 8), 
    Type = rep(c("n.atrisk", "n.event"), each = 4), Category = rep(c("Placebo", 
        "Low GES", "Medium GES", "High GES"), 2), rbind(n.atrisk, 
        n.event))
plot(KM, fun = "event", col = c("black", "red", "blue", 
    "purple4"), lty = c(1, 1, 1, 1), xlab = "Months since the week 26 visit", 
    ylab = "", lwd = 4.7, axes = F, xlim = c(0, 40), mark.time = TRUE)
# str(V1)
legend(0, 0.0075, c("Placebo", "Low GES", "Medium GES", 
    "High GES"), lty = c(1, 1, 1, 1), lwd = c(2, 2, 2), 
    col = c("black", "red", "blue", "purple4"), bty = "n", 
    cex = 0.8, y.intersp = 0.8)
box(bty = "l")
axis(1, at = seq(0, 40, by = 12), label = c("0", "12", "24", 
    "36"))
axis(2, at = seq(0, 0.01, by = 0.002), label = c("0.000", 
    "0.002", "0.004", "0.006", "0.008", " 0.010"))
mtext(side = 1, at = -10, text = "Number at risk:", line = 4, 
    cex = 1, adj = 0)
mtext(side = 1, at = -10, text = "Placebo", line = 5, cex = 1, 
    adj = 0)
mtext(side = 1, at = -10, text = "Low GES", line = 6, cex = 1, 
    adj = 0)
mtext(side = 1, at = -10, text = "Medium GES", line = 7, 
    cex = 1, adj = 0)
mtext(side = 1, at = -10, text = "High GES", line = 8, cex = 1, 
    adj = 0)

mtext(side = 1, text = n.atrisk[1, ], line = 5, at = ptime, 
    cex = 1)
mtext(side = 1, text = n.atrisk[2, ], line = 6, at = ptime, 
    cex = 1)
mtext(side = 1, text = n.atrisk[3, ], line = 7, at = ptime, 
    cex = 1)
mtext(side = 1, text = n.atrisk[4, ], line = 8, at = ptime, 
    cex = 1)

mtext(side = 1, text = paste("(", n.event[1, ], ")"), line = 5, 
    at = ptime + 3, cex = 0.9)
mtext(side = 1, text = paste("(", n.event[2, ], ")"), line = 6, 
    at = ptime + 3, cex = 0.9)
mtext(side = 1, text = paste("(", n.event[3, ], ")"), line = 7, 
    at = ptime + 3, cex = 0.9)
mtext(side = 1, text = paste("(", n.event[4, ], ")"), line = 8, 
    at = ptime + 3, cex = 0.9)

mtext(side = 2, "Probability of acquiring HIV-1 infection", 
    cex = 1.1, outer = T, las = 3, line = 1)
```

#### 4.3. Fig. 2C

Vaccine efficacy (VE) for the GES subgroups versus the entire placebo group was estimated as one minus the odds of infection in vaccine recipients with Low/Medium/High response divided by the odds of infection in the entire placebo group HIV-1 negative at week 24 of enrollment in the study.

```
flrtime.vp <- dat.vcc.allplc_3$time_to_infect - dat.vcc.allplc_3$time_to_wk26
flrstatus.vp <- ifelse(dat.vcc.allplc_3$infect == "Yes", 
    1, 0)
sex.vp <- ifelse(dat.vcc.allplc_3$dem_sex == "Female", 1, 
    0)
risk.cat.vp <- ifelse(dat.vcc.allplc_3$BRA_risk == "Low", 
    0, ifelse(dat.vcc.allplc_3$BRA_risk == "Medium", 1, 
        2))
risk.medium.vp <- ifelse(dat.vcc.allplc_3$BRA_risk == "Medium", 
    1, 0)
risk.high.vp <- ifelse(dat.vcc.allplc_3$BRA_risk == "High", 
    1, 0)

############# two-phase design ################
nn0.vp <- c(length(dat1$pin[dat1$infect == "No" & dat1$cc_cohort == 
    1 & dat1$trt == "VACCINE" & dat1$dem_sex == "Female" & 
    dat1$perprot == "Yes" & dat1$vaccno == 4]), length(dat1$pin[dat1$infect == 
    "No" & dat1$cc_cohort == 1 & dat1$trt == "VACCINE" & 
    dat1$dem_sex == "Male" & dat1$perprot == "Yes" & dat1$vaccno == 
    4]), length(dat1$pin[dat1$infect == "No" & dat1$cc_cohort == 
    1 & dat1$trt == "VACCINE" & dat1$dem_sex == "Female" & 
    dat1$perprot == "No" & dat1$vaccno == 4]), length(dat1$pin[dat1$infect == 
    "No" & dat1$cc_cohort == 1 & dat1$trt == "VACCINE" & 
    dat1$dem_sex == "Male" & dat1$perprot == "No" & dat1$vaccno == 
    4]), length(dat1$pin[dat1$infect == "No" & dat1$cc_cohort == 
    1 & dat1$trt == "VACCINE" & dat1$dem_sex == "Female" & 
    dat1$perprot == "No" & dat1$vaccno < 4]), length(dat1$pin[dat1$infect == 
    "No" & dat1$cc_cohort == 1 & dat1$trt == "PLACEBO" & 
    (dat1$time_to_infect > dat1$time_to_wk26)]))


nn1.vp <- c(length(dat1$pin[dat1$infect == "Yes" & dat1$cc_cohort == 
    1 & dat1$trt == "VACCINE" & dat1$dem_sex == "Female" & 
    dat1$perprot == "Yes" & dat1$vaccno == 4]), length(dat1$pin[dat1$infect == 
    "Yes" & dat1$cc_cohort == 1 & dat1$trt == "VACCINE" & 
    dat1$dem_sex == "Male" & dat1$perprot == "Yes" & dat1$vaccno == 
    4]), length(dat1$pin[dat1$infect == "Yes" & dat1$cc_cohort == 
    1 & dat1$trt == "VACCINE" & dat1$dem_sex == "Female" & 
    dat1$perprot == "No" & dat1$vaccno == 4]), length(dat1$pin[dat1$infect == 
    "Yes" & dat1$cc_cohort == 1 & dat1$trt == "VACCINE" & 
    dat1$dem_sex == "Male" & dat1$perprot == "No" & dat1$vaccno == 
    4]), length(dat1$pin[dat1$infect == "Yes" & dat1$cc_cohort == 
    1 & dat1$trt == "VACCINE" & dat1$dem_sex == "Female" & 
    dat1$perprot == "No" & dat1$vaccno < 4]), length(dat1$pin[dat1$infect == 
    "Yes" & dat1$cc_cohort == 1 & dat1$trt == "PLACEBO" & 
    (dat1$time_to_infect > dat1$time_to_wk26)]))


stratuminds.vp <- ifelse(dat.vcc.allplc_3$trt == "VACCINE" & 
    dat.vcc.allplc_3$dem_sex == "Female" & dat.vcc.allplc_3$perprot == 
    "Yes" & dat.vcc.allplc_3$vaccno == 4, 1, ifelse(dat.vcc.allplc_3$trt == 
    "VACCINE" & dat.vcc.allplc_3$dem_sex == "Male" & dat.vcc.allplc_3$perprot == 
    "Yes" & dat.vcc.allplc_3$vaccno == 4, 2, ifelse(dat.vcc.allplc_3$trt == 
    "VACCINE" & dat.vcc.allplc_3$dem_sex == "Female" & dat.vcc.allplc_3$perprot == 
    "No" & dat.vcc.allplc_3$vaccno == 4, 3, ifelse(dat.vcc.allplc_3$trt == 
    "VACCINE" & dat.vcc.allplc_3$dem_sex == "Male" & dat.vcc.allplc_3$perprot == 
    "No" & dat.vcc.allplc_3$vaccno == 4, 4, ifelse(dat.vcc.allplc_3$trt == 
    "VACCINE" & dat.vcc.allplc_3$dem_sex == "Female" & dat.vcc.allplc_3$perprot == 
    "No" & dat.vcc.allplc_3$vaccno < 4, 5, 6)))))


fit.tps.vp.cat63 <- tps(flrstatus.vp ~ factor(new) + sex.vp + 
    risk.medium.vp + risk.high.vp, nn0 = nn0.vp, nn1 = nn1.vp, 
    group = stratuminds.vp, method = "ML", cohort = TRUE, 
    data = dat.vcc.allplc_3)
```

Vaccine efficacy plot showing that VE increased significantly in individuals with high GES.

```
out1 <- fit.tps.vp.cat63$coef
out2 <- diag(fit.tps.vp.cat63$covm)


I = 2
q = t(fit.tps.vp.cat63$coef[I]) %*% solve(fit.tps.vp.cat63$covm[c(I), 
    c(I)]) %*% fit.tps.vp.cat63$coef[I]
p.one = 1 - pchisq(q, 1)
I = 3
q = t(fit.tps.vp.cat63$coef[I]) %*% solve(fit.tps.vp.cat63$covm[c(I), 
    c(I)]) %*% fit.tps.vp.cat63$coef[I]
p.two = 1 - pchisq(q, 1)

I = 4
q = t(fit.tps.vp.cat63$coef[I]) %*% solve(fit.tps.vp.cat63$covm[c(I), 
    c(I)]) %*% fit.tps.vp.cat63$coef[I]
p.three = 1 - pchisq(q, 1)
par(mar = c(3.5, 5, 2.1, 2.1), bty = "l")

plotCI(x = 1 - exp(out1[2:4]), ui = 1 - exp(out1[2:4] - 
    1.96 * sqrt(out2[2:4])), li = 1 - exp(out1[2:4] + 1.96 * 
    sqrt(out2[2:4])), col = c("red", "blue", "purple4"), 
    barcol = c("red", "blue", "purple4"), lwd = 3, xaxt = "n", 
    yaxt = "n", xlab = "", ylab = "", xlim = c(0.25, 3.55), 
    ylim = c(-2, 1), cex.lab = 1.4, cex.main = 1.4)


axis(2, at = seq(-2, 1, by = 0.5), labels = paste(seq(-200, 
    100, by = 50), "%", sep = ""), lwd.ticks = 2, tcl = -0.6, 
    las = 2, cex.axis = 1.1)
axis(2, at = seq(-2, 1, by = 0.1), labels = NA, cex.axis = 1.1, 
    tcl = -0.4)
axis(side = 1, at = 1:3, labels = c("Low", "Medium", "High"), 
    cex.axis = 1.1)

abline(h = 0, lty = 2, col = 2)
title(ylab = "Estimated VE", cex.lab = 1.2, line = 4)
title(xlab = "GES", cex.lab = 1.2, line = 2.2)

pvals = sapply(c(p.one, p.two, p.three), sprintf, fmt = "p = %0.2f")
text(x = 1:3, y = rep(0.98, 1), pvals)
text(x = c(1.3, 2.3, 3.3), y = 1 - exp(out1[2:4]), paste(round(100 * 
    (1 - exp(out1[2:4])), 2), "%", sep = ""))
text(x = c(1.3, 2.3, 3.3), y = 1 - exp(out1[2:4] - 1.96 * 
    sqrt(out2[2:4])), paste(round(100 * (1 - exp(out1[2:4] - 
    1.96 * sqrt(out2[2:4]))), 2), "%", sep = ""))
text(x = c(1.3, 2.3, 3.3), y = 1 - exp(out1[2:4] + 1.96 * 
    sqrt(out2[2:4])), paste(round(100 * (1 - exp(out1[2:4] + 
    1.96 * sqrt(out2[2:4]))), 2), "%", sep = ""))
```

#### 4.4. Fig. 2D

The dataset was randomly split into training and testing sets in a 7:3 ratio, while retaining class distributions within the groups. A logistic regression of GES was fit on to the training dataset (Prentice et al., 2015). The model’s discriminative ability was evaluated by generating a receiver operator characteristic curve (ROC) and the corresponding AUC on the test dataset. The prediction accuracy of the model was also assessed on the test dataset. The probability that gives minimum mis-classification error was chosen as the cut-off. This process was repeated 1000 times.

```
######## repeat data partition 1000: caculate accuracy & auc
######## #######

auc <- NULL
p_auc <- NULL
k <- 1000
acc <- NULL
cutp <- NULL
pbar <- create_progress_bar("text")
pbar$init(k)

set.seed(123)

for (i in 1:k) {
    # Train-test splitting 70% of samples -> training 30% of
    # samples -> testing
    trainIndex <- createDataPartition(mydata_rv144_vac_enriched63_170$infect, 
        p = 0.7, list = FALSE)
    train <- mydata_rv144_vac_enriched63_170[trainIndex, 
        ]
    test <- mydata_rv144_vac_enriched63_170[-trainIndex, 
        ]

    flrstatus <- ifelse(test$infect == "Yes", 1, 0)
    sex <- ifelse(test$dem_sex == "Female", 1, 0)
    risk.cat <- ifelse(test$BRA_risk == "Low", 0, ifelse(test$BRA_risk == 
        "Medium", 1, 2))
    risk.medium <- ifelse(test$BRA_risk == "Medium", 1, 
        0)
    risk.high <- ifelse(test$BRA_risk == "High", 1, 0)
    EIGEN1 <- test$EIGEN1
    trans63 = test$trans_63
    test_data = as.data.frame(cbind(flrstatus, sex, risk.medium, 
        risk.high, EIGEN1, trans63))
    rownames(test_data) = rownames(test)

    flrstatus <- ifelse(train$infect == "Yes", 1, 0)
    sex <- ifelse(train$dem_sex == "Female", 1, 0)
    risk.cat <- ifelse(train$BRA_risk == "Low", 0, ifelse(train$BRA_risk == 
        "Medium", 1, 2))
    risk.medium <- ifelse(train$BRA_risk == "Medium", 1, 
        0)
    risk.high <- ifelse(train$BRA_risk == "High", 1, 0)
    EIGEN1 <- train$EIGEN1
    trans63 = train$trans_63
    train_data = as.data.frame(cbind(flrstatus, sex, risk.medium, 
        risk.high, EIGEN1, trans63))
    rownames(train_data) = rownames(train)

    model <- glm(flrstatus ~ trans63 + EIGEN1 + sex + risk.medium + 
        risk.high, family = binomial(link = "logit"), data = train_data)

    cutp <- optimalCutoff(actuals = train_data$flrstatus, 
        predictedScores = predict(model, train_data, type = "response"), 
        optimiseFor = "misclasserror")

    results_prob <- predict(model, test_data, type = "response")

    results <- ifelse(results_prob > cutp, 1, 0)

    answers <- test_data$flrstatus

    misClasificError <- mean(answers != results)

    acc[i] <- 1 - misClasificError
    auc[i] = roc.area(answers, results_prob)$A

    pbar$step()
}
```

Histogram and density curve of AUC and accuracy is plotted.

The distribution of AUC and accuracy plotted after repeating the process 1000 times showed that GES could predict HIV-1 infection with AUC of 0.67 ± 0.08 and with accuracy of 0.81 ± 0.04.

```
hist(auc, col = "green4", border = "green4", density = 70, 
    probability = TRUE, xlim = c(0.3, 1), ylim = c(0, 15), 
    xlab = "", breaks = 30, main = "")
par(new = TRUE)
plot(density(acc, bw = 0.02), xlim = c(0.3, 1), ylim = c(0, 
    15), main = "", xlab = "AUC & Accuracy", col = "orange", 
    lwd = 2, bty = "n")
legend(x = 0.9, y = 15, "AUC", fill = "green4", density = 70, 
    border = "green4", bty = "n", cex = 0.8)
legend(x = 0.9, y = 14, "Accuracy", col = "orange", lty = 1, 
    lwd = 2, seg.len = 1, bty = "n", cex = 0.8)
```

### 5. Strong relationship between functional ADCP responses in a human vaccine trial and the protective RV144 signature

GSEA analyses for comparing the magnitude of ADCP in groups categorized into high and low, from two different timepoints at day 3 (visit 5B) and week 2 (visit 6) after last vaccination. The results of the GSE29618\_BCELL\_VS\_MONOCYTE\_DAY7\_FLU\_VACCINE\_DN gene signature was imported for analysis.

```
# Reading in GSEA result folders
v5Bwk26ADCP <- read.table(file = "Data/v5B_ADCP_wk26_method_2_GT.Gsea.1592331997827/GSE29618_BCELL_VS_MONOCYTE_DAY7_FLU_VACCINE_DN.xls", 
    sep = "\t", header = T)
v6wk26ADCP <- read.table(file = "Data/v6_ADCP_wk26_method_2_GT.Gsea.1592331997828/GSE29618_BCELL_VS_MONOCYTE_DAY7_FLU_VACCINE_DN.xls", 
    sep = "\t", header = T)

# Reading in GSEA run statistics
gsea_results <- read_xlsx("Data/Updated_core_enrichment.xlsx", 
    sheet = 2)
```

Statistics for the genesets (p value, NES, etc) as well as enrichment genes within the geneset were generated.

#### 5.1. Fig. 3A

Plotting the gene enrichment for the week 2 and day 3 timepoints.

```
# Setting plot labels

x_lab = paste("←", "ADCP High        Rank in Ordered Dataset       ADCP Low", 
    "→")
y_lab = "Enrichment Score (ES)"


### Updated Enrichment Plots (combined Day 3 + Week 2)

# Creating legends for Day 3 (v5B)

nes_v5Bwk26ADCP = signif(gsea_results %>%
    filter(analysis == "v5B_ADCP_wk26_GT") %>%
    dplyr::select(NES), digits = 2)

pval_v5Bwk26ADCP = ifelse(round(gsea_results %>%
    filter(analysis == "v5B_ADCP_wk26_GT") %>%
    dplyr::select(NOM.p.val), digits = 3) == 0, 0.001, round(gsea_results %>%
    filter(analysis == "v5B_ADCP_wk26_GT") %>%
    dplyr::select(NOM.p.val), digits = 3))

name_v5Bwk26ADCP = "Day 3"
legend_v5Bwk26ADCP = paste(name_v5Bwk26ADCP, " NES = ", 
    nes_v5Bwk26ADCP, ", p < ", pval_v5Bwk26ADCP, sep = "")
max_v5Bwk26ADCP <- max(v5Bwk26ADCP$RANK.IN.GENE.LIST)


# Creating legends for Week 2 (v6)

nes_v6wk26ADCP = signif(gsea_results %>%
    filter(analysis == "v6_ADCP_wk26_GT") %>%
    dplyr::select(NES), digits = 2)

pval_v6wk26ADCP = ifelse(round(gsea_results %>%
    filter(analysis == "v6_ADCP_wk26_GT") %>%
    dplyr::select(NOM.p.val), digits = 3) == 0, 0.001, round(gsea_results %>%
    filter(analysis == "v6_ADCP_wk26_GT") %>%
    dplyr::select(NOM.p.val), digits = 3))

name_v6wk26ADCP = "Week 2"
legend_v6wk26ADCP = paste(name_v6wk26ADCP, " NES = ", nes_v6wk26ADCP, 
    ", p < ", pval_v6wk26ADCP, sep = "")
max_v6wk26ADCP <- max(v6wk26ADCP$RANK.IN.GENE.LIST)


# Plotting ES v/s Rank

plotmax <- ifelse(max_v5Bwk26ADCP > max_v6wk26ADCP, max_v5Bwk26ADCP, 
    max_v6wk26ADCP)

tplot1 <- ggplot(data = v6wk26ADCP, aes(x = RANK.IN.GENE.LIST, 
    y = RUNNING.ES)) + geom_line(aes(color = "#2844c9"), 
    size = 1) + geom_line(data = v5Bwk26ADCP, mapping = aes(x = RANK.IN.GENE.LIST, 
    y = RUNNING.ES, color = "red"), size = 1) + labs(x = x_lab, 
    y = y_lab, color = "Legend\n") + scale_color_manual(labels = c(legend_v6wk26ADCP, 
    legend_v5Bwk26ADCP), values = c("#2844c9", "red")) + 
    theme_bw()

tplot3 <- tplot1 + theme(panel.grid.major = element_blank(), 
    panel.grid.minor = element_blank(), panel.background = element_blank()) + 
    theme(axis.title.y = element_text(colour = "black", 
        size = 16, family = "Arial"), axis.title.x = element_text(colour = "black", 
        size = 16, family = "Arial"), axis.line = element_line(color = "black"), 
        axis.text.x = element_text(color = "black", size = "14", 
            family = "Arial"), axis.text.y = element_text(color = "black", 
            size = "14", family = "Arial"), panel.border = element_blank(), 
        panel.background = element_blank()) + geom_hline(yintercept = 0, 
    color = "black") + theme(legend.position = "bottom", 
    legend.title = element_blank(), plot.margin = unit(c(1, 
        1, 1, 1), "cm"), legend.text = element_text(color = "black", 
        size = "14", family = "Arial")) + xlim(0, plotmax) + 
    ylim(-0.3, 0.7) + theme(legend.justification = c(1, 
    1), legend.position = c(1, 1), legend.box.margin = margin(rep(2, 
    4)))
```

#### 5.2. Fig. 3B

The model built using ADCP GES from day 3 was able to predict ADCP responses measured 2 weeks after the last vaccination with an accuracy of 0.71. The receiver operator characteristic (ROC) curve illustrates the discriminating ability of the classifier from the day 3 training dataset (AUC = 0.8, 95% CI: 0.6 to 0.99, p = 0.01) and the week 2 testing dataset (AUC = 0.73, 95% CI: 0.5 to 0.95, p = 0.03) to predict ADCP responses.

```
load("Data/RV306 ADCP GES.RData")
mydata_rv306_v5b_ADCP_wk26$y = ifelse(mydata_rv306_v5b_ADCP_wk26$adcpGT_wk26_vac_cat_updated == 
    "Low", 1, 0)
mydata_rv306_v6_ADCP_wk26$y = ifelse(mydata_rv306_v6_ADCP_wk26$adcpGT_wk26_vac_cat_updated == 
    "Low", 1, 0)

mylogit = glm(y ~ trans_93, data = mydata_rv306_v5b_ADCP_wk26, 
    family = binomial(link = "logit"))
prob = predict(mylogit, type = c("response"), newdata = mydata_rv306_v5b_ADCP_wk26)
pred = prediction(prob, mydata_rv306_v5b_ADCP_wk26$y)
perf = performance(pred, "tpr", "fpr")
par(mar = c(5.1, 5.1, 5.1, 5.1), bty = "l", family = "sans")
plot(perf, col = "red", xlab = "1-Specificity", ylab = "Sensitivity", 
    font.axis = 2, lwd = 2, main = " ")
abline(0, 1, lwd = 2)
auc = performance(pred, "auc")
auc = round(unlist(slot(auc, "y.values")), 2)
y_obs = mydata_rv306_v5b_ADCP_wk26$y
probpred = predict(mylogit, mydata_rv306_v5b_ADCP_wk26, 
    type = "response")
p = roc.area(y_obs, probpred)$p.value
ci = ci.auc(mydata_rv306_v5b_ADCP_wk26$y, mydata_rv306_v5b_ADCP_wk26$trans_93)

legend(0.5, 0.4, c(paste("Training (Day 3)"), paste("AUC = ", 
    round(auc, 2))), cex = 0.9, text.col = "red", bty = "n")
### v6 #####
prob <- predict(mylogit, mydata_rv306_v6_ADCP_wk26, type = "response")
pred = prediction(prob, mydata_rv306_v6_ADCP_wk26$y)
perf = performance(pred, "tpr", "fpr")
par(new = TRUE)
plot(perf, col = "blue", xlab = "", ylab = "", lwd = 2)

auc = performance(pred, "auc")
auc = round(unlist(slot(auc, "y.values")), 3)
y_obs = mydata_rv306_v6_ADCP_wk26$y
probpred = predict(mylogit, mydata_rv306_v6_ADCP_wk26, type = "response")
p = roc.area(y_obs, probpred)$p.value
ci = ci.auc(mydata_rv306_v6_ADCP_wk26$y, mydata_rv306_v6_ADCP_wk26$trans_93)
legend(0.5, 0.2, c(paste("Test (Week 2)"), paste("AUC = ", 
    round(auc, 2))), cex = 0.9, text.col = "blue", bty = "n")
```

#### 5.3. Fig. 3C

Correlation plot showing that GES computed from the enriched genes associating with ADCP correlated strongly with the protective GES in the RV144 study.

```
load("Data/mydata_rv144_GES_cor.RData")

trans63 = mydata_rv144_GES_cor$trans_63
trans91 = mydata_rv144_GES_cor$trans_91
trans115 = mydata_rv144_GES_cor$trans_115

##### CORRELATION & PLOT ########## TRANS91 ####


myplot = as.data.frame(cbind(trans63, trans91))
p91 <- ggplot(myplot, aes(x = trans63, y = trans91)) + geom_point() + 
    xlab("GES Infection") + ylab("GES ADCP Day 3") + theme_bw() + 
    theme(text = element_text(family = "Arial"), axis.text = element_text(color = "black", 
        size = 12), axis.title = element_text(size = 14), 
        panel.border = element_blank(), panel.grid.major = element_blank(), 
        panel.grid.minor = element_blank(), axis.line = element_line(colour = "black")) + 

stat_smooth(formula = y ~ x, method = "lm", col = "#C42126", 
    se = FALSE, size = 1)

### TRANS115 ####
myplot = as.data.frame(cbind(trans63, trans115))
p115 <- ggplot(myplot, aes(x = trans63, y = trans115)) + 
    geom_point() + xlab("GES Infection") + ylab("GES ADCP Week 2") + 
    theme_bw() + theme(text = element_text(family = "Arial"), 
    axis.text = element_text(color = "black", size = 12), 
    axis.title = element_text(size = 14), panel.border = element_blank(), 
    panel.grid.major = element_blank(), panel.grid.minor = element_blank(), 
    axis.line = element_line(colour = "black")) + 
stat_smooth(formula = y ~ x, method = "lm", col = "#C42126", 
    se = FALSE, size = 1)
```

Plotting the correlation at the week 2 timepoint.

```
p115 + annotate("text", x = 0.7, y = 1.4, label = "Rho = 0.74   p = 2.2e-16", 
    col = "black", family = "Arial", size = 4.5)
```

#### 5.4. Fig. 3D

Plotting the correlation at the day 3 timepoint.

```
p91 + annotate("text", x = 0.7, y = 1.3, label = "Rho = 0.75   p = 2.2e-16", 
    col = "black", family = "Arial", size = 4.5)
```

### 6. Overlapping enriched genes associating with ADCP response

#### 6.1. Fig. 4A

Overlapping genes between the day 3 and week 2 timepoints were computed and displayed.

```
enriched_genes = read_xlsx("Data/Gene_lists.xlsx", sheet = 1)

names(enriched_genes)[c(5, 6)] = c("Day 3", "Week 2")
enriched_gene_lists = as.list(enriched_genes)

# Removing NA from Day 3 list, caused due to difference
# in number of row
enriched_gene_lists$`Day 3` = na.omit(enriched_gene_lists$`Day 3`)

# Plotting a Venn diagram with required colors
ggvenn(enriched_gene_lists, c("Day 3", "Week 2"), show_percentage = FALSE, 
    text_size = 6, fill_color = c("red", rgb(10, 100, 200, 
        max = 255)), fill_alpha = 0.85)
```

#### 6.2. Fig. 4B

A GES was computed using the **82** genes overlapping between the 118 enriched genes from week 2 and the 93 enriched genes from day 3 post the RV144 vaccine regimen that associated with ADCP. The model using GES obtained from the 82 genes was also able to predict ADCP responses measured 2 weeks after vaccination with an accuracy of 0.71. The ROC curve illustrates the discriminating ability of the classifier from the day 3 training dataset (AUC = 0.81, 95%CI: 0.62 to 1, p = 0.007) and the week 2 testing dataset (AUC = 0.75, 95%CI: 0.53 to 0.97, p = 0.02) to predict ADCP responses.

```
load("Data/RV306 ADCP GES overlap.RData")
mydata_rv306_v5b_ADCP_wk26_overlap82$y = ifelse(mydata_rv306_v5b_ADCP_wk26_overlap82$adcpGT_wk26_vac_cat_updated == 
    "Low", 1, 0)
mydata_rv306_v6_ADCP_wk26_overlap82$y = ifelse(mydata_rv306_v6_ADCP_wk26_overlap82$adcpGT_wk26_vac_cat_updated == 
    "Low", 1, 0)

mylogit = glm(y ~ trans_82, data = mydata_rv306_v5b_ADCP_wk26_overlap82, 
    family = binomial(link = "logit"))
prob = predict(mylogit, type = c("response"), newdata = mydata_rv306_v5b_ADCP_wk26_overlap82)
pred = prediction(prob, mydata_rv306_v5b_ADCP_wk26_overlap82$y)
perf = performance(pred, "tpr", "fpr")
par(mar = c(5.1, 5.1, 5.1, 5.1), bty = "l", family = "sans")
plot(perf, col = "red", xlab = "1-Specificity", ylab = "Sensitivity", 
    font.axis = 2, lwd = 2, main = " ")
abline(0, 1, lwd = 2)
auc = performance(pred, "auc")
auc = round(unlist(slot(auc, "y.values")), 2)
y_obs = mydata_rv306_v5b_ADCP_wk26_overlap82$y
probpred = predict(mylogit, mydata_rv306_v5b_ADCP_wk26_overlap82, 
    type = "response")
p = roc.area(y_obs, probpred)$p.value
ci = ci.auc(mydata_rv306_v5b_ADCP_wk26_overlap82$y, mydata_rv306_v5b_ADCP_wk26_overlap82$trans_82)

legend(0.5, 0.4, c(paste("Training (Day 3)"), paste("AUC = ", 
    round(auc, 2))), cex = 0.9, text.col = "red", bty = "n")
### v6 #####
prob <- predict(mylogit, mydata_rv306_v6_ADCP_wk26_overlap82, 
    type = "response")
pred = prediction(prob, mydata_rv306_v6_ADCP_wk26_overlap82$y)
perf = performance(pred, "tpr", "fpr")
par(new = TRUE)
plot(perf, col = "blue", xlab = "", ylab = "", lwd = 2)

auc = performance(pred, "auc")
auc = round(unlist(slot(auc, "y.values")), 3)
y_obs = mydata_rv306_v6_ADCP_wk26_overlap82$y
probpred = predict(mylogit, mydata_rv306_v6_ADCP_wk26_overlap82, 
    type = "response")
p = roc.area(y_obs, probpred)$p.value
ci = ci.auc(mydata_rv306_v6_ADCP_wk26_overlap82$y, mydata_rv306_v6_ADCP_wk26_overlap82$trans_82)
legend(0.5, 0.2, c(paste("Test (Week 2)"), paste("AUC = ", 
    round(auc, 2))), cex = 0.9, text.col = "blue", bty = "n")
```

#### 6.3. Fig. 4C

Heatmap of the 82 overlapping genes enriched at both timepoints with the ADCP phenotype, stratified by high or low ADCP magnitude.

```
# Determining overlapping genes between timepoints and
# creating heatmap Reading in gene expression matrices
v6_data = read.table(file = "Data/RV306_v6_forADCP_GSEA.txt", 
    header = T, sep = "\t")
v5B_data = read.table(file = "Data/RV306_v5B_forADCP_GSEA.txt", 
    header = T, sep = "\t")

enriched_genes = read_xlsx("Data/Gene_lists.xlsx", sheet = 1)

names(enriched_genes)[c(5, 6)] = c("Day 3", "Week 2")
enriched_gene_lists = as.list(enriched_genes)

# Removing NA from Day 3 list, caused due to difference
# in number of row
enriched_gene_lists$`Day 3` = na.omit(enriched_gene_lists$`Day 3`)


overlap_genes = as.data.frame(intersect(enriched_genes$`Day 3`, 
    enriched_genes$`Week 2`))
names(overlap_genes)[1] = "Overlapping_genes"

v6_data = v6_data[-c(1)]
names(v6_data)[1] = "Gene_name"
v6_data = right_join(v6_data, overlap_genes, by = c(Gene_name = "Overlapping_genes"))
rownames(v6_data) = v6_data$Gene_name
v6_data[1] = NULL

v5B_data = v5B_data[-c(1)]
names(v5B_data)[1] = "Gene_name"
v5B_data = right_join(v5B_data, overlap_genes, by = c(Gene_name = "Overlapping_genes"))
rownames(v5B_data) = v5B_data$Gene_name
v5B_data[1] = NULL

# Reading in metadata containing sample classification
# information
metadata = read_xlsx("Data/Gene_lists.xlsx", sheet = 2)
meta_v5B = subset(metadata, Timepoint == "v5B")
meta_v6 = subset(metadata, Timepoint == "v6")

# Subsetting samples only to those with ADCP
v5B_data = v5B_data %>%
    dplyr::select(meta_v5B$Sample_ID)
v6_data = v6_data %>%
    dplyr::select(meta_v6$Sample_ID)

combined_set = cbind(v5B_data, v6_data)

# Calculating mean of expression values for each
# category

combined_set$Day_3_ADCP_High = rowMeans(combined_set[, 1:12])
combined_set$Day_3_ADCP_Low = rowMeans(combined_set[, 13:21])
combined_set$Week_2_ADCP_High = rowMeans(combined_set[, 
    22:35])
combined_set$Week_2_ADCP_Low = rowMeans(combined_set[, 36:45])

average_counts = as.matrix(combined_set[-c(1:45)])


### Using this matrix to create heatmap


# Setting a color scale
orig_col_scale = colorRampPalette(c("white", "orange", "#721427"))(n = 299)

# Created a dataframe categorizing matrix columns by
# group Used for color annotation

color_df = read.table(file = "Data/Color_DF.txt", header = T, 
    sep = "\t", row.names = "Name")

# Assigning colors to categories
annot_colors = list(Timepoint = c(`Day 3` = "#12752C", `Week 2` = "#F58142"), 
    ADCP = c(High = "#882B8D", Low = "#5AC4F7"))

# Plotting the heatmap
p1 = pheatmap(average_counts, color = orig_col_scale, scale = "row", 
    cluster_rows = T, cluster_cols = F, clustering_method = "complete", 
    cellheight = 8, cellwidth = 60, annotation_col = color_df, 
    annotation_colors = annot_colors, annotation_legend = T, 
    annotation_names_col = T, border_color = NA, show_colnames = F, 
    fontsize_row = 7, display_numbers = F, labels_row = as.expression(lapply(rownames(average_counts), 
        function(a) bquote(italic(.(a))))))
```

```
p2 = ggplotify::as.ggplot(p1) + annotate("text", x = 0.72, 
    y = 0.985, label = "Z-score", fontface = 2, size = 3.5) + 
    theme(panel.background = element_rect(fill = "white", 
        linetype = "blank"))

p2
```

#### 6.4. Fig. 4D

The list of **82** genes enriched with ADCP were uploaded in GeneMANIA. Edges are based on physical interactions, co-expression, co-localization, and shared pathways. Circles depict the **82** genes, gold circles are the four genes that belong to the gene ontology phagocytosis pathway, blue circles are genes that are directly connected to them, diamonds indicate related pathways, and the color of the edge indicates the type of connection.

Source data for the plots: Gene\_lists.xlsx

### 7. Pathway analyses of the enriched genes in the different vaccine studies

#### 7.1. Fig. 5A & B

The 178 genes enriched with ACDP or infection status in any of the nine analyses, as well as the 82 genes overlapping in enrichment between the two RV306 timepoints, were used as search terms in GeneMANIA in the Cytoscape software. We selected connections such as co-expression, co-localization, pathway, and physical interactions, as well as Reactome and MSigDB for attributes. Zero additional genes and up to 10 additional attributes were found with GO biological process-based weighting. The genes in the nine-analysis network waswere clustered further using the MCODE algorithm in the clusterMaker2 Cytoscape plugin with default settings.

Source data for the plots: Gene\_lists.xlsx

#### 7.2. Fig. 5C

Pathway analyses was performed using Metascape with default parameters.

https://metascape.org/gp/index.html#/main/step1

Source data for the plots: Gene\_lists.xlsx

### 8. Cellular origin of the RV144 signature

The Seurat object can be created from the rds and metadata files provided. The rds file included are the normalized cell surface protein feature barcoding (FB) and gene (mRNA) expression matrices and the embeddings for the tSNE created from the feature barcoding data. Metadata for the sample and clustering results for each cell and sample metadata with the ADCP group assignment is provided.

Load the data and metadata:

```
FB_norm = readRDS("Data/10x_FB_norm.rds")
RNA_norm = readRDS("Data/10x_RNA_norm.rds")
tsne_FB_embeddings = readRDS("Data/10x_tsne_embed.rds")
integrated_metadata = readRDS("Data/10x_cell_meta.rds")
sample_metadata = read.table("Data/10x_sample_metadata.txt", 
    stringsAsFactors = FALSE, sep = "\t", header = TRUE)
```

The Seurat object is assembled and scaled.

```
integrated = CreateSeuratObject(RNA_norm)
integrated[["FB"]] = CreateAssayObject(data = FB_norm)
integrated$sample = as.character(integrated_metadata$sample)
integrated$dist_snn_res.0.5 = as.character(integrated_metadata$dist_snn_res.0.5)
integrated$ADCP_status = recode(integrated$sample, !!!set_names(sample_metadata$ADCP_status, 
    sample_metadata$PID))
integrated[["tsne_FB"]] = CreateDimReducObject(embeddings = tsne_FB_embeddings, 
    key = "FBtSNE_", assay = "FB")
integrated = ScaleData(integrated, verbose = FALSE)
```

The clusters are annotated, and the expression of the 63 gene RV144 signature in the different cell types are used to order the cell types for plotting.

```
integrated$prot_annot = recode(integrated$dist_snn_res.0.5, 
    `0` = "CD4+ naive T", `1` = "CD4+ memory T", `2` = "CD8+ naive T", 
    `3` = "CD8+ memory T", `4` = "CD56dim NK", `5` = "CD14+ monocytes", 
    `6` = "Naive B cells", `7` = paste0("V", "γ", "9/V", 
        "δ", "2 T cell"), `8` = "CD16+ monocytes", `9` = "Megakaryocytes", 
    `10` = "MAIT", `11` = "Memory B cells", `12` = "mDC", 
    `14` = "CD56hi NK", `15` = "pDC", `16` = "Plasmablasts")

gene_list_63 = c("PXN", "PTGER2", "SERINC5", "LRPAP1", "SEMA4A", 
    "PKM", "MPP1", "LEPROT", "LMNA", "TKT", "AP2S1", "PSAP", 
    "MYO1F", "GAA", "CD14", "CTSD", "LGALS2", "GRN", "ALDH3B1", 
    "AMPD2", "PGD", "GAS7", "IL17RA", "TIMP2", "ZDHHC7", 
    "ZNF467", "CD68", "TBXAS1", "TYROBP", "FCGRT", "RNF130", 
    "CST3", "C5AR1", "DUSP6", "MAFB", "HMOX1", "CSF1R", 
    "TBC1D8", "IMPA2", "NPL", "CAMKK2", "CTBP2", "PYGL", 
    "QPCT", "RAB20", "RBM47", "NAGA", "SCARB2", "DAPK1", 
    "CEBPA", "SIRPA", "SIRPB1", "ARRB1", "SLCO3A1", "SORT1", 
    "SULT1A2", "CD163", "KIAA0513", "ADAM9", "CCR1", "FZD1", 
    "SLC36A1", "FLVCR2")

Idents(integrated) = "prot_annot"
avg_exp_63 = AverageExpression(integrated, features = gene_list_63, 
    assay = "RNA", verbose = FALSE)$RNA
hclust_63_genes = hclust(dist(as.matrix(avg_exp_63)))
hclust_63_cells = hclust(dist(t(as.matrix(avg_exp_63))))

celltype_order = hclust_63_cells$labels[hclust_63_cells$order]
gene_order = hclust_63_genes$labels[hclust_63_genes$order]


integrated$prot_annot = factor(integrated$prot_annot, levels = celltype_order)
```

The cell subset percentages for each donor were calculated, and cell subsets with average percentages > 1% were selected.

```
cell_type_pops = as.data.frame.matrix(table(integrated$prot_annot, 
    integrated$sample))
donor_percents = map_df(cell_type_pops, ~prop.table(.) * 
    100)
donor_percents = as.data.frame(t(donor_percents))
colnames(donor_percents) = rownames(cell_type_pops)
group_info = distinct(data.frame(group = integrated$ADCP_status, 
    sample = integrated$sample))
donor_percents$group = recode(rownames(donor_percents), 
    !!!set_names(group_info$group, group_info$sample))
average_percents = donor_percents %>%
    select(-group) %>%
    t() %>%
    as.data.frame() %>%
    rowMeans()
pops_to_keep = names(average_percents)[average_percents > 
    1]
```

The Seurat object is split into smaller objects for each cell type, and differential gene expression was run to compare the cells from high and low ADCP groups of individuals. The results can be filtered to exclude genes that are not protein coding or that have been selected by type for exclusion (e.g. RPS, MT genes.)

```
cell_subsets = SplitObject(integrated, split.by = "prot_annot")

source("Code/convenience_functions.R")
cell_subsets_ADCP_deg = map(cell_subsets, doFindMarkers, 
    ident.1 = "high", ident.2 = "low", logfc.threshold = 0.25, 
    min.pct = 0.1, group.by = "ADCP_status", abs_logFC = TRUE)

genes_exclude = scan("Data/10x_genes_exclude.txt", what = "c")

ADCP_deg = bind_rows(cell_subsets_ADCP_deg, .id = "cell_type") %>%
    filter(!gene %in% genes_exclude, cell_type %in% pops_to_keep)
```

#### 8.1. Fig. 6A

Dimensionality reduction tSNE plot of the different cell clusters in the single cell RV306 dataset.

```
p1 = DimPlot(integrated, group.by = "prot_annot", pt.size = 1, 
    cols = pals::glasbey()[-c(4, 6)], label = TRUE, repel = TRUE, 
    reduction = "tsne_FB") + coord_fixed() + labs(x = "tSNE_1", 
    y = "tSNE_2") + theme(axis.title = element_text(size = 11), 
    axis.text = element_text(size = 9)) + scale_x_continuous(breaks = seq(-25, 
    30, by = 5)) + scale_y_continuous(breaks = seq(-25, 
    30, by = 5))
```

#### 8.2. Fig. 6B

Heatmap of the 63 gene signature from the single cell RV306 dataset

```
p1 = DoHeatmap(integrated, features = gene_order, group.by = "prot_annot", 
    group.bar = TRUE, group.colors = pals::glasbey()[-c(4, 
        6)], label = FALSE, draw.lines = FALSE) + theme(text = element_text(size = 16), 
    axis.text.y = element_text(size = 12, face = "italic", 
        color = "black"), legend.text = element_text(size = 14))
```

#### 8.3. Fig. 6C

Logistic regression that accounted for the sampling design was used for the univariate analyses of the 63 enriched genes. A radar plot of the significant genes was generated to illustrate odds ratios (OR) and 95% confidence intervals. All ORs were reported per 1-SD increase. All logistic regression models were adjusted for gender and baseline risk behavior and one significant principal component axis (Haynes et al., 2012; Prentice et al., 2015).

```
flrstatus <- ifelse(mydata_rv144_vac_enriched63_170$infect == 
    "Yes", 1, 0)
sex <- ifelse(mydata_rv144_vac_enriched63_170$dem_sex == 
    "Female", 1, 0)
risk.cat <- ifelse(mydata_rv144_vac_enriched63_170$BRA_risk == 
    "Low", 0, ifelse(mydata_rv144_vac_enriched63_170$BRA_risk == 
    "Medium", 1, 2))
risk.medium <- ifelse(mydata_rv144_vac_enriched63_170$BRA_risk == 
    "Medium", 1, 0)
risk.high <- ifelse(mydata_rv144_vac_enriched63_170$BRA_risk == 
    "High", 1, 0)
EIGEN1 <- mydata_rv144_vac_enriched63_170$EIGEN1

xmat = data.frame(scale(mydata_rv144_vac_enriched63_170[, 
    1:63]))
ORmat_tps = matrix(NA, ncol(xmat), 5)
colnames(ORmat_tps) = c("Gene", "OR", "95%CI LB", "95%CI UB", 
    "p value")
for (i in 1:ncol(xmat)) {
    x = xmat[, i]
    fit.tps <- tps(flrstatus ~ x + EIGEN1 + sex + risk.medium + 
        risk.high, nn0 = nn0, nn1 = nn1, group = stratuminds, 
        method = "ML", cohort = TRUE)
    ORmat_tps[i, 1] = colnames(xmat[i])

    ORmat_tps[i, 2:5] = as.matrix(cbind(round(exp(fit.tps$coef[2]), 
        2), round(exp(fit.tps$coef[2] - sqrt(fit.tps$covm[2, 
        2]) * 1.96), 2), round(exp(fit.tps$coef[2] + sqrt(fit.tps$covm[2, 
        2]) * 1.96), 2), round(min(2 * (1 - pnorm(abs(fit.tps$coef[2]/sqrt(fit.tps$covm[2, 
        2])))), 1), 4)))
}

ORmat_tps = as.data.frame(ORmat_tps)
ORmat_tps = unfactor(ORmat_tps)
pval = as.numeric(ORmat_tps[, 5])
fdr = round(p.adjust(pval, method = "fdr"), 4)
result = cbind(ORmat_tps, fdr)
result_p005 = result[which(result$`p value` < 0.05 & result$fdr < 
    0.1), ]
```

Radar plot of OR for genes with p < 0.05 & fdr < 0.1

```
mydata = result_p005
rownames(mydata) = mydata$Gene
mydata = mydata[, 2:4]
mydata = t(mydata)
mydata = as.data.frame(mydata)
mydata = rbind(rep(1, 32), rep(0, 32), mydata)

op <- par(mar = c(1, 1, 1, 1), mfrow = c(1, 1), family = "Arial")

radarchart(mydata[, c(1, 32:2)], axistype = 4, centerzero = TRUE, 
    pty = c(16, 32, 32), plty = c(1, 4, 3), plwd = c(2, 
        2, 2), pcol = c("purple3", "grey40", "grey20"), 
    cglcol = "grey", cglty = 1, axislabcol = "grey20", cglwd = 0.7, 
    calcex = 0.65, vlcex = 0.65, title = "", seg = 5, caxislabels = c("0", 
        "0.2", "0.4", "0.6", "0.8", "1"))

par(xpd = TRUE)
legend(x = -1.2, y = -1.25, legend = c("OR"), bty = "n", 
    lty = c(1), pch = c(16), lwd = c(2), seg.len = 3.5, 
    col = c("purple3"), text.col = c("purple3"), cex = 0.7, 
    pt.cex = 1, text.font = 2, y.intersp = 1.7)

legend(x = -0.4, y = -1.25, legend = c("95%CI UB"), bty = "n", 
    lty = c(3), pch = c(NA), lwd = c(2), seg.len = 3.5, 
    col = c("grey20"), text.col = c("grey20"), cex = 0.7, 
    pt.cex = 1, text.font = 2, y.intersp = 1.7)

legend(x = 0.5, y = -1.25, legend = c("95%CI LB"), bty = "n", 
    lty = c(4), pch = c(NA), lwd = c(2), seg.len = 3.5, 
    col = c("grey30"), text.col = c("grey30"), cex = 0.7, 
    pt.cex = 1, text.font = 2, y.intersp = 1.7)
```

#### 8.4. Fig. 6D

tSNE plots showing gene expression of specific 7 genes of interest.

```
feats_RV144 = c("SEMA4A", "IL17RA", "CTSD", "CD68", "GAA", 
    "SLC36A1", "SERINC5")
p1 = FeaturePlot(integrated, features = feats_RV144, ncol = 3, 
    reduction = "tsne_FB", combine = FALSE, order = TRUE)
p1 = map(p1, ~.x + coord_fixed() + labs(x = "tSNE_1", y = "tSNE_2") + 
    NoLegend())
```

#### 8.5. Fig. 6E

Barplot of significant DEGs between clusters of cell subsets comparing vaccinated individuals with high or low ADCP. A threshold of adjusted p value < 0.05 was the criteria for significance. CD14+ monocytes are highlighted in red in the barplot and the cell subsets are sorted by number of significant differentially expressed genes.

```
ADCP_barplot = ADCP_deg %>%
    filter(p_val_adj < 0.05, cell_type %in% pops_to_keep) %>%
    group_by(cell_type) %>%
    count() %>%
    mutate(color = ifelse(cell_type == "CD14+ monocytes", 
        "red", "blue"))
ADCP_barplot$cell_type = factor(ADCP_barplot$cell_type, 
    levels = ADCP_barplot %>%
        arrange(-n) %>%
        pull(cell_type))
```

```
p1 = ADCP_barplot %>%
    ggplot(mapping = aes(x = cell_type, y = n, fill = color)) + 
    geom_col() + scale_fill_manual(values = c(blue = "blue", 
    red = "red")) + labs(x = NULL, y = "# DEG") + theme_classic() + 
    theme(axis.title = element_text(size = 20), axis.text = element_text(size = 14, 
        color = "black"), axis.text.x = element_text(angle = 90, 
        vjust = 0.5, hjust = 1), axis.line = element_blank(), 
        axis.ticks = element_blank()) + scale_y_continuous(limits = c(0, 
    65), expand = c(0, 0)) + NoLegend()
```

```
sessionInfo()
```

```
## R version 3.6.1 (2019-07-05)
## Platform: x86_64-apple-darwin15.6.0 (64-bit)
## Running under: macOS Mojave 10.14.6
## 
## Matrix products: default
## BLAS:   /Library/Frameworks/R.framework/Versions/3.6/Resources/lib/libRblas.0.dylib
## LAPACK: /Library/Frameworks/R.framework/Versions/3.6/Resources/lib/libRlapack.dylib
## 
## Random number generation:
##  RNG:     Mersenne-Twister 
##  Normal:  Inversion 
##  Sample:  Rounding 
##  
## locale:
## [1] en_US.UTF-8/en_US.UTF-8/en_US.UTF-8/C/en_US.UTF-8/en_US.UTF-8
## 
## attached base packages:
## [1] grid      stats     graphics  grDevices utils     datasets  methods  
## [8] base     
## 
## other attached packages:
##  [1] ggplotify_0.0.9        pheatmap_1.0.12        readxl_1.3.1          
##  [4] ggvenn_0.1.9           Seurat_3.1.1           huxtable_5.3.0        
##  [7] cowplot_1.1.1          forcats_0.5.1          stringr_1.4.0         
## [10] dplyr_1.0.5            purrr_0.3.4            readr_1.4.0           
## [13] tidyr_1.1.3            tibble_3.1.1           tidyverse_1.3.1       
## [16] extrafont_0.17         plyr_1.8.6             pROC_1.17.0.1         
## [19] ROCR_1.0-11            verification_1.42      dtw_1.22-3            
## [22] proxy_0.4-25           CircStats_0.2-6        MASS_7.3-51.4         
## [25] boot_1.3-22            fields_11.6            spam_2.6-0            
## [28] dotCall64_1.0-1        caret_6.0-86           InformationValue_1.2.3
## [31] fmsb_0.7.0             survival_3.2-11        forestplot_1.10.1     
## [34] checkmate_2.0.0        magrittr_2.0.1         knitr_1.33            
## [37] ggplot2_3.3.3          lattice_0.20-44        varhandle_2.0.5       
## [40] rockchalk_1.8.144      gplots_3.1.1           osDesign_1.8          
## 
## loaded via a namespace (and not attached):
##   [1] utf8_1.2.1           reticulate_1.20      R.utils_2.10.1      
##   [4] tidyselect_1.1.1     lme4_1.1-26          htmlwidgets_1.5.3   
##   [7] Rtsne_0.15           munsell_0.5.0        codetools_0.2-16    
##  [10] mutoss_0.1-12        ica_1.0-2            statmod_1.4.35      
##  [13] future_1.21.0        withr_2.4.2          colorspace_2.0-1    
##  [16] Biobase_2.46.0       highr_0.9            rstudioapi_0.13     
##  [19] stats4_3.6.1         Rttf2pt1_1.3.8       listenv_0.8.0       
##  [22] labeling_0.4.2       Rdpack_2.1.1         mnormt_2.0.2        
##  [25] farver_2.1.0         parallelly_1.25.0    vctrs_0.3.8         
##  [28] generics_0.1.0       TH.data_1.0-10       ipred_0.9-11        
##  [31] xfun_0.22            R6_2.5.0             rsvd_1.0.3          
##  [34] pals_1.7             gridGraphics_0.5-1   bitops_1.0-7        
##  [37] assertthat_0.2.1     SDMTools_1.1-221     scales_1.1.1        
##  [40] multcomp_1.4-17      nnet_7.3-12          gtable_0.3.0        
##  [43] globals_0.14.0       sandwich_3.0-0       timeDate_3043.102   
##  [46] rlang_0.4.11         splines_3.6.1        lazyeval_0.2.2      
##  [49] extrafontdb_1.0      ModelMetrics_1.2.2.2 dichromat_2.0-0     
##  [52] broom_0.7.6          yaml_2.2.1           reshape2_1.4.4      
##  [55] modelr_0.1.8         backports_1.2.1      tools_3.6.1         
##  [58] lava_1.6.9           ellipsis_0.3.2       RColorBrewer_1.1-2  
##  [61] BiocGenerics_0.32.0  ggridges_0.5.3       TFisher_0.2.0       
##  [64] Rcpp_1.0.6           rpart_4.1-15         pbapply_1.4-3       
##  [67] zoo_1.8-9            haven_2.4.1          ggrepel_0.9.1       
##  [70] cluster_2.1.0        fs_1.5.0             data.table_1.13.6   
##  [73] openxlsx_4.2.3       lmtest_0.9-38        reprex_2.0.0        
##  [76] RANN_2.6.1           tmvnsim_1.0-2        mvtnorm_1.1-1       
##  [79] matrixStats_0.58.0   fitdistrplus_1.1-3   hms_1.0.0           
##  [82] evaluate_0.14        xtable_1.8-4         gridExtra_2.3       
##  [85] compiler_3.6.1       maps_3.3.0           KernSmooth_2.23-15  
##  [88] crayon_1.4.1         minqa_1.2.4          R.oo_1.24.0         
##  [91] htmltools_0.5.1.1    mgcv_1.8-28          lubridate_1.7.10    
##  [94] DBI_1.1.1            formatR_1.9          kutils_1.70         
##  [97] dbplyr_2.1.1         Matrix_1.2-17        cli_2.5.0           
## [100] R.methodsS3_1.8.1    rbibutils_2.1.1      parallel_3.6.1      
## [103] metap_1.4            gower_0.2.2          igraph_1.2.6        
## [106] pkgconfig_2.0.3      sn_2.0.0             numDeriv_2016.8-1.1 
## [109] foreign_0.8-71       plotly_4.9.3         recipes_0.1.16      
## [112] xml2_1.3.2           foreach_1.5.1        multtest_2.42.0     
## [115] prodlim_2019.11.13   rvest_1.0.0          yulab.utils_0.0.2   
## [118] digest_0.6.27        tsne_0.1-3           sctransform_0.3.2   
## [121] RcppAnnoy_0.0.18     rmarkdown_2.7        cellranger_1.1.0    
## [124] leiden_0.3.7         uwot_0.1.10          gtools_3.8.2        
## [127] nloptr_1.2.2.2       lifecycle_1.0.0      nlme_3.1-140        
## [130] jsonlite_1.7.2       carData_3.0-4        mapproj_1.2.7       
## [133] viridisLite_0.4.0    fansi_0.4.2          pillar_1.6.0        
## [136] httr_1.4.2           plotrix_3.8-1        glue_1.4.2          
## [139] zip_2.1.1            png_0.1-7            iterators_1.0.13    
## [142] class_7.3-15         stringi_1.5.3        caTools_1.18.2      
## [145] mathjaxr_1.4-0       irlba_2.3.3          future.apply_1.7.0  
## [148] ape_5.5
```
